# The complete mitochondrial genome of *Graphium phidias* (Papilionoidea; Papilionidae; Papilioninae)

**DOI:** 10.1128/mra.00487-26

**Published:** 2026-09-21

**Authors:** Rui Han, Xun Bian, Bin Zhang

**Affiliations:** 1 College of Life Sciences & Technology, Inner Mongolia Normal University 71203, Hohhot, China; 2 Key Laboratory of Ecology of Rare and Endangered Species and Environmental Protection (Guangxi Normal University), Ministry of Education 12388, Guilin, China; 3 Key Laboratory of Biodiversity Conservation and Sustainable Utilization for College and University of Inner Mongolia Autonomous Region, Hohhot, China; University of California Riverside, Riverside, California, USA

**Keywords:** mitogenome, China, *Graphium*

## Abstract

*Graphium phidias* (Oberthür, 1906) is a butterfly insect classified under the subgenus *Paranticopsis*, genus *Graphium*. It is a double-stranded circular structure with a total length of 15,059 bp, and the A + T content is 79.2%. In this study, the complete mitochondrial genome sequence of *Graphium phidias* was obtained and annotated to fill the gap in genomic data of *Graphium phidias* and provides important molecular evidence for research on the phylogeny, evolution, and mitochondrial function of Lepidoptera.

## ANNOUNCEMENT

*Graphium phidias* (Oberthür, 1906) belongs to the subgenus *Paranticopsis* within the genus *Graphium* of Papilioninae and is distributed in Vietnam and China (Guangxi, Guizhou, etc.). Currently, the mitochondrial genome of this species has not been characterized or reported in the literature, and only morphological descriptions of this species were available (1–3). Although the NCBI database has archived mitochondrial genome data for numerous Papilionidae species, the mitochondrial genome of *G. phidias* has not yet been reported.

The specimen of *Graphium phidias* was collected from Libo, Guizhou, and preserved in absolute ethanol. It was stored in a −20°C ultra-low temperature refrigerator at Guangxi Normal University. Total DNA was extracted from the muscle tissue of the hind femur following the manufacturer’s instructions for the Tiangen DNeasy Blood & Tissue Kit. The quality and concentration of the extracted DNA were evaluated by agarose gel electrophoresis. Qualified DNA samples were sent to BGI Genomics Co., Ltd. (Shenzhen), where raw data were generated using the DNBSEQ sequencing platform with a paired-end 150 bp (PE150) sequencing strategy. Raw reads (16.7 Gb) were obtained and filtered to generate clean data with fastp v.0.20.0 (4), which were stored in FASTQ format. The high-throughput sequencing data were compared with the mitochondrial genome sequences of closely related species available in the NCBI database. The sequence with the highest homology to the clean reads, *Graphium chironides* (OP973781), was selected as the reference sequence. Subsequently, the mitochondrial genome was assembled using NOVOPlasty v.4.2.1 (5). Parameters were set in the configuration file config.txt as follows: Type=mito, Genome Range=14000–18000, K-mer=39, Max memory=16. Assembly was executed with the command: “perl NOVOPlasty4.2.1.pl -c config.txt” (6, 7). The obtained mitochondrial genome was annotated using the online service of MITOS (http://mitos.bioinf.uni-leipzig.de/index.py) (8). Manual annotation was also performed using MEGA software (9).

**Fig 1:**
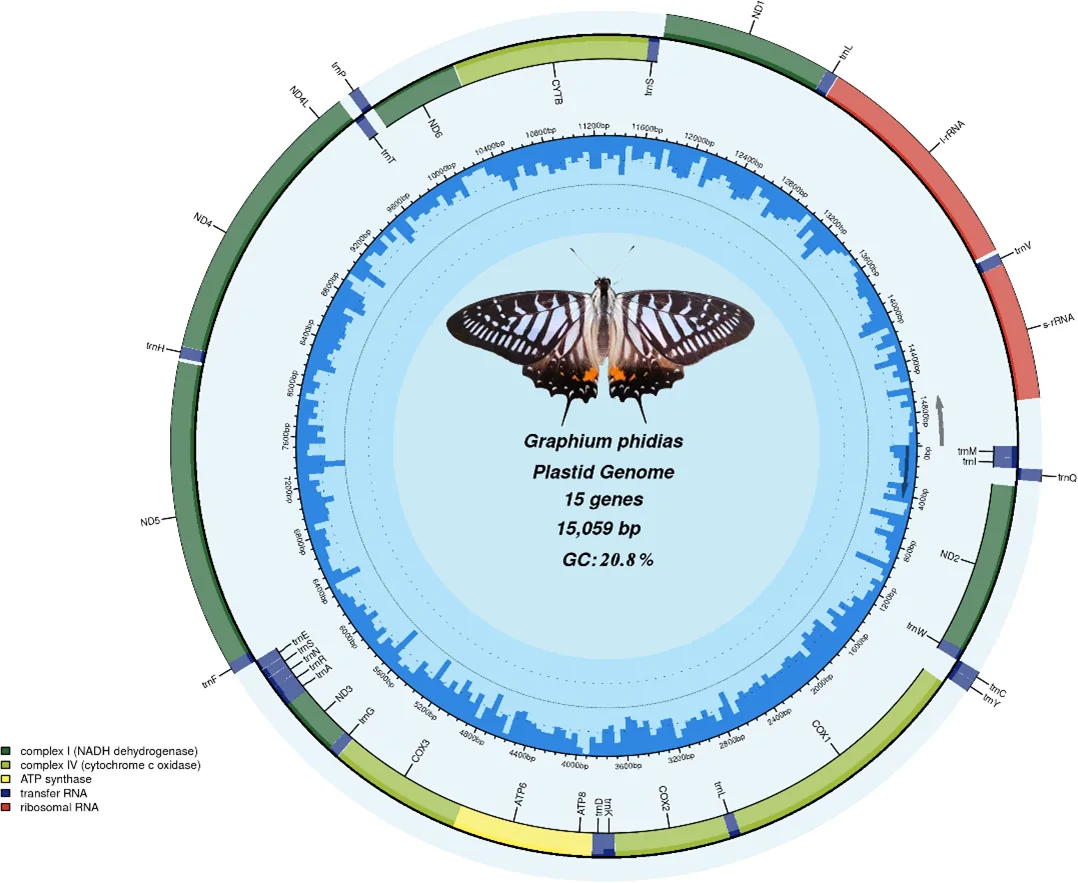
Mitochondrial genome map of *Graphium phidias*. The genome was annotated via MITOS and plotted with Chloroplot v.0.2.4 (10). Different colors denote functional gene categories, as indicated in the figure. The innermost ring shows GC content, with a total length of 15,059 bp.

The mitochondrial genome sequence of *Graphium phidias* has been deposited in the GenBank database with the accession number PV788242.1. It is a double-stranded circular molecule with a total length of 15,059 bp (Fig. 1), and the G + C content is 20.8%. The mitochondrial genome consists of 13 protein-coding genes (PCGs), 22 transfer RNA (tRNA) genes, 2 ribosomal RNA (rRNA) genes, and 1 non-coding region. All 13 PCGs employ ATN (ATT, ATG) as the initiation codon and TAN (TAA, TAG) or incomplete T as the termination codon. A total of 22 tRNA genes were identified, with their lengths ranging from 59 bp (*trns1*) to 71 bp (*trnK*). The rRNA genes consist of *rrnS* (767 bp) and *rrnL* (1,303 bp), which are the two core ribosomal RNA components of the mitogenome (Table 1).

**TABLE 1:** Annotation of the mitochondrial genome of *Graphium phidias*

Name	Start	Stop	Direction	Length	ovl/nc	Codons
*trnM(cat*)	1	67	F	67	0	
*trnI(gat*)	68	131	F	64	−3	
*trnQ(ttg*)	129	197	R	69	38	
*nad2*	236	1249	F	1,014	−2	ATT/TAA
*trnW(tca*)	1248	1314	F	67	−7	
*trnC(gca*)	1307	1368	R	62	0	
*trnY(gta*)	1369	1432	R	64	35	
*cox1*	1468	2965	F	1,498	0	ATG/TAA
*trnL2(taa*)	2966	3032	F	67	0	
*cox2*	3033	3714	F	682	0	ATG/T
*trnK(ctt*)	3715	3785	F	71	−1	
*trnD(gtc*)	3785	3851	F	67	0	
*atp8*	3852	4013	F	162	−7	ATT/TAA
*atp6*	4007	4684	F	678	−1	ATG/TAA
*cox3*	4684	5472	F	789	3	ATG/TAA
*trnG(tcc*)	5476	5541	F	66	0	
*nad3*	5542	5895	F	354	−2	ATT/TAG
*trnA(tgc*)	5894	5957	F	64	−1	
*trnR(tcg*)	5957	6021	F	65	0	
*trnN(gtt*)	6022	6086	F	65	1	
*trnS1(gct*)	6088	6146	F	59	1	
*trnE(ttc*)	6148	6213	F	66	−2	
*trnF(gaa*)	6212	6277	R	66	−1	
*nad5*	6277	7998	R	1,722	18	ATT/TAA
*trnH(gtg*)	8017	8083	R	67	1	
*nad4*	8085	9425	R	1,341	0	ATG/TAA
*nad4l*	9426	9713	R	288	2	ATG/TAA
*trnT(tgt*)	9716	9779	F	64	0	
*trnP(tgg*)	9780	9844	R	65	2	
*nad6*	9847	10377	F	531	6	ATT/TAA
*cob*	10384	11544	F	1,161	−2	ATG/TAA
*trnS2(tga*)	11543	11608	F	66	17	
*nad1*	11626	12564	R	939	1	ATG/TAG
*trnL1(tag*)	12566	12632	R	67	5	
*rrnL*	12638	13940	R	1,303	22	
*trnV(tac*)	13963	14026	R	64	0	
*rrnS*	14027	14793	R	767	0	

## Data Availability

The complete mitochondrial genome sequence of *Graphium phidias* is available in GenBank under the accession number PV788242.1. The corresponding BioProject, BioSample, and SRA numbers are PRJNA1477715, SAMN60767390, and SRR30081743, respectively.
